# Effect of retirement on medical reimbursement expenses—evidence from China

**DOI:** 10.1186/s13561-023-00434-x

**Published:** 2023-04-13

**Authors:** Yuanyang Wu, Jiahui Pang, Hualei Yang

**Affiliations:** 1 School of Medicine and Health Management, Tongji Medical College, Huazhong University of Science and Technology, Wuhan, 430030 Hubei China; 2 School of Public Administration, Zhongnan University of Economics and Law, Wuhan, 430073 China

**Keywords:** Fuzzy regression discontinuity, Retirement, Medical reimbursement expenses, Transmission mechanism

## Abstract

**Background:**

Medical reimbursement in China is not for all diseases, and medical reimbursement expenses are not completely consistent with medical service demand, though the scope and proportion of medical reimbursement are gradually expanding. This study aimed to examine the effect of retirement on medical reimbursement expenses in urban China.

**Methods:**

The effect of retirement on medical reimbursement expenses were estimated by using fuzzy regression discontinuity based on data from the China Health and Retirement Longitudinal Study (CHARLS) in 2011, 2013, 2015, and 2018. Its group heterogeneity by educational backgrounds and marital status, and underlying mechanisms were also explored.

**Results:**

Retirement increased medical reimbursement expenses of outpatient significantly (*P* < 0.05).Low time cost and deteriorating health status after retirement were possible mechanisms in this association. Retirement increased the reimbursement expenses significantly among the older adults with more educational obtainment or being widowed/divorced.

**Conclusion:**

The above findings indicated that there was a positive association between retirement and medical reimbursement expenses. The scope and proportion of medical reimbursement should be incorporated into retirement policy for improving medical service accessibility and rational healthcare utilization of retired population.

**Supplementary Information:**

The online version contains supplementary material available at 10.1186/s13561-023-00434-x.

## Introduction

Aging population issue gradually become a major challenge for China’s social development. According to national seventh census, there were 264 million people aged 60 and above, accounting for about 18.7% of the total population in China. Accompanied by the extension of life expectancy, retired population was increasing rapidly. The “baby boom” from 1962 to 1975 exceeded 25 million in China, and it was expected that more than 22 million people was going to retire each year nowadays.

Retirement occurred in the middle and later stages of life. Current Chinese retirement policy indicated that statutory retirement age was 60 for male workers, 50 for female workers, and 55 for female cadres. In China, the old-age stage often was longevous but sick, which raised the concerns about health status and healthcare utilization of retired population [5]. On the one hand, based on the intergenerational perspective, the large scale of retired population led to less income and more expenditures due to non-payment policy of retirement group, increasing the payment pressure and affecting the structural stability of the health insurance fund. On the other hand, retirement mostly occurred in the old age when physical functions were declining. Retired population in poor health had more medical service demands, but medical reimbursement expenses were not completely consistent with medical service demands. Therefore, there was a huge gap between medical reimbursement and medical service demand for retired population in China.

Most studies estimated the effect of retirement on the sustainability of health insurance fund from a national level [21, 22, 24], and further focused on the association between retirement on individual healthcare utilization and medical service demand, such as Zhang et al. [23], Wei and Zhou [19] and He et al. [9]. However, there were few studies exploring the relationship between retirement and medical reimbursement expenses. Medical reimbursement expenses referred to the expenses paid through the medical insurance fund, and were obtained by subtracting out-of-pocket expenses from the total medical expenses, which was different from healthcare utilization focusing on the consumption of medical services by medical service consumers or patients in the medical service market. In China, there was a huge gap between medical reimbursement and healthcare utilization due to non-reimbursement policy for some diseases and medical services. The reimbursement expenses were closely associated with *Medical Insurance Catalog*, including drugs list, diagnosis and treatment items list, and medical service facilities list. The patients can reimburse their medical expenses according to the prescribed proportion after receiving medical services within the scope of three lists. Compared to healthcare utilization, estimating the effect of retirement on medical reimbursement expenses better reflected the choice or propensity of retirees to use medical reimbursement services when receiving medical services, such as, using more drugs in *Medical Insurance Catalog*. And the transmission mechanisms behind healthcare utilization and medical reimbursement were different. Healthcare utilization was associated with medical demands, but medical reimbursement was achieved through receiving more medical services within the *Medical Insurance Catalog.*

Therefore, this study aimed to estimate the effect of retirement on medical reimbursement expenses of Chinese urban retired population based on the data from the China Health and Retirement Longitudinal Study (CHARLS) in 2011, 2013, 2015, and 2018 by using fuzzy regression discontinuity (FRD) and further explored group heterogeneity under different educational backgrounds and marital status, and its underlying mechanisms.

## Research background and literature review

Unlike the retirement system in many developed countries, China implements a mandatory retirement system, but there are different arrangements for different kinds of employees. As workers, males are to retire at the age of 60 and females at the age of 50, and as cadres, males can retire at the age of 60 and females at the age of 55. For employees, people who are engaged in special types of work such as high-risk work or work that is harmful to health, men can retire at 55 years old, and women can retire at 45 years old; for employees who have work-related illnesses and are incapacitated, both men and women can retire early, at 50 and 40 years old, respectively.

Current basic medical insurance system in China mainly comprises two types: Urban Employee’s Basic Medical Insurance (UEBMI) and Urban and Rural Resident Basic Medical Insurance (URRBMI). In the 1950s, China established a labor insurance system and a free medical care, providing basic medical services for urban workers. In 1998, the “Decision of the State Council on Establishing the Basic Medical Insurance System for Urban Employees” was promulgated and the UEBMI replaced the labor insurance system.^1^ However, at that time, the rural cooperative medical coverage rate was only 6.5%. Therefore, establishing a new rural cooperative medical system to ensure the health of rural residents became an urgent matter. Subsequently, in 2003, the “Opinions on Establishing a New Rural Cooperative Medical System” was issued, and a new rural cooperative medical system (NCMS) was established, which was organized, guided, and supported by the government, and funded by individuals, collectives, and the government, focusing on the major diseases treatment.^2^ In 2016, the basic medical insurance system for urban and rural residents was established by integrating the NCMS with the Urban Resident Basic Medical Insurance (URBMI).^3^

At the same time, the basic medical insurance system continued to expand its coverage and improve actual reimbursement ratio. The coverage rate increased from about 15% in 2000 to nearly 95% by the end of 2010, covering a total of 1.27 billion people. The actual reimbursement ratio of UEBMI for hospitalization expenses gradually increased from 71.2% in 2007 to 82.1% in 2014. The actual reimbursement ratio of the NCMS for hospitalization expenses increased from 43% in 2010 to 56.6% in 2014. The actual reimbursement ratio of URBMI for hospitalization expenses increased from less than 50% in 2007 to 66.5% in 2014. After integrating the NCMS with the URBMI, the actual reimbursement ratio of hospitalization expenses within the basic medical insurance system for urban and rural residents was 70.0% in 2020, compared to 65.6% in 2018, an increase of 4.4% in two years [10].

Most European and American studies supported the positive effect of retirement on healthcare utilization. Lucifora and Vigani [14] found that retirement increased the number of outpatient care and the probability of outpatient visits based on SHARE data from ten European countries. Similarly with the study by Bíró [2], retirement impelled a 3% to 10% growth of outpatient visits in Europe. More leisure time and stronger health preferences promoted medical service utilization. In addition, in terms of drug use, Puig-Junoy et al. [16] found that reimbursement from health insurance increased drug consumption by an average of 17.5% and total drug expenditures by 25% by using Differences-in-Differences method. Boaz and Muller [3] found that retirement increased the likelihood of physician services and the number of visits based on data from the Longitudinal Retirement History Survey (RHS) in the United States.

There were similar findings in the Asian studies. Dang [4] found that retirement significantly increased outpatient utilization based on data from Vietnamese towns. In Japan, Tokuda et al. [17] found that only the unemployed elderly aged 70 ~ 74 had a higher outpatient care rate. Kananurak [11] used a structured questionnaire to survey 400 Thai social health insurance participants and found that the poor health, the high-income and the retirees with chronic disease tended to use more healthcare measures. In China, Zhang et al. [23] found that retirement increased out-of-pocket expenses for self-treatment by $177 per month, and retirement had a significantly positive effect on the number of outpatient visits and hospitalizations. He et al. [9] also verified the positive effect of retirement on medical service utilization.

The transmission mechanism was possibly three channels, including low time cost, changes of socioeconomic status and deteriorating health status. First, individuals were liberated from their stressful and strenuous work after retirement, and received more medical services due to more leisure time. When working, a leave for outpatient care meant loss of income and unemployment risk, therefore, patients might delay their treatment, especially for hospitalization. After retirement, the time cost for medical services decreased. There was no worry about income loss and unemployment risk [9, 23]. The second possible explanation was change of socioeconomic status. In Chinese context, the socioeconomic status after retirement decreased dramatically, especially for those who were key leadership when working. As the Chinese saying goes, the house was crowded with visitors when at work, the house was deserted when at retirement. The changes of psychological feelings might be especially prominent for males, because it was often difficult for males to re-established their new social identities after retirement [18]. The third possible explanation was health status. Zou et al. [27] found that after retirement, urban residents’ tobacco consumption dropped by about 30 ~ 40%, and their alcohol consumption also showed a significant decline based on the Urban Household Survey(UHS), resulting in changes of health status.

## Methods

### Data sources

China Health and Retirement Longitudinal Study (CHARLS) was selected for this study, which was a high-quality microscopic data of households and individuals aged 45 and above in China. It was a reliable data for empirical analysis of China’s aging population. More than 600 SCI, CSSCI, and CSCD literatures used CHARLS data to analyze aging issues from 2016 in China National Knowledge Infrastructure (CNKI). Therefore, CHARLS data in 2011, 2013, 2015 and 2018 were selected for our analysis. First, 77,223 individuals were surveyed in the four surveys, which was a large sample for empirical analysis. Second, the data contained both baseline data from 2011 and latest data in 2018, which excluded the bias of period effects of cross-sectional data. In addition, we focused on individual age to 55 ~ 65, and finally kept 30,899 samples after eliminating missing values and outliers.

### Variables

#### Reimbursement expenses

Reimbursement expenses of outpatient, inpatient and medicine were dependent variables, that was the reimbursement part by health insurance from total medical expenditures. The reimbursement part was obtained by subtracting the out-of-pocket expenses from the total medical expenditures. The reimbursement expenses of outpatient were the reimbursement part from total medical expenditures of outpatient during the last month, reimbursement expenses of inpatient were the reimbursement part from total medical expenditures of inpatient care during the past year, and reimbursement expenses of medicine were the reimbursement part from total expenditures for purchasing medicine during the last month. All reimbursement expenses were measured by Yuan and in logarithmic form.

#### Retirement

Retirement was independent variable, and was defined by self-reporting “processed retirement procedures”. According to the questionnaire “Have you carried out retirement procedures? As a reminder, retirement was defined as retirement from governmental departments, institutions and enterprises, as well as retirement of flexibly employed persons who had participated in basic pension insurance.” A dummy variable for retirement was generated based on the respondents’ responses, with 1 for retired and 0 for unretired.

#### Control variables

Individual-level and family-level factors were selected as control variables after referring to previous studies of Zhang et al. [23] and He et al. [9]. Therefore, years of education, marital status, living with children, household assets, and living area (urban–rural) were selected as control variables.

#### Mechanism variables

Time cost and health status were selected to verify the transmission mechanism in the effect of retirement on reimbursement expenses. Therefore, outpatient incidence, outpatient times, and working hours were selected to estimate the effect of time cost. (1) Outpatient incidence: a dummy indicating whether the respondent used outpatient care in the past month or not. (2) Outpatient times: times of outpatient care in the past month. (3)Working hours: a dummy indicating whether working hours more than 40 h per week, with 1: more than 40 h;0:lesss than 40 h. (4) Number of chronic diseases: the number of chronic diseases the respondents had. (5)Activities of Daily living: a dummy indicating whether the respondent could independently complete six daily behaviors, with 1:not;0:yes.(6) Self-rated health status: self-reported health status on a scale from 1 to 5, with 1:poor; 2:fair; 3: good; 4: very good; 5: excellent.(7)CES-D scores: individuals’ scores of Center for Epidemiological Studies Depression Scale from 0 ~ 30. (8)BMI index: a dummy variable of Body Mass Index, with 1 for overweight and 0 for not.

### Empirical strategy

This study aimed to estimate the effect of retirement on medical reimbursement expenses, namely,

1 $${H}_{i}={\beta }_{0}+{\beta }_{1}{R}_{i}+{\varepsilon }_{i}$$

*R*_*i*_ denotes the retirement status of individual *i* and *H*_*i*_ is the individual’s medical imbursement expenses. When estimating the effect, the results were biased by endogenous retirement status, as a negative health shock could induce an individual to retire. To address this, a fuzzy regression discontinuity (FRD) approach was used to identify this association, leveraging the threshold in eligibility for retirement at the age of 60. FRD equation was as follows,

2 $${H}_{a}={\alpha }_{0}+{\alpha }_{1}{R}_{a}+{\alpha }_{2}{Age}_{a}+{\alpha }_{3}{Age}_{a}^{2}+{\alpha }_{4}{X}_{a}+{\varepsilon }_{a}$$

where ${R}_{a}$ is a dummy indicating if an individual is older than the age required to be eligible for retirement. ${Age}_{a}$ indicates individual’s age and ${Age}_{a}^{2}$ is the square of individual’s age. ${X}_{a}$ was control variables, including marital status, education attainment and others. ${\varepsilon }_{a}$ was random error. The validity test was performed to ensure the validity of results when using FRD: first, assignment variable could not be manipulated. The individual’s age was randomly distributed near the breakpoint, and the age was continuous at the breakpoint. Second, the predetermined variables could not make significant jumps at the breakpoints. It was impossible to determine whether the effect at the breakpoints were caused by the core explanatory variables or other variables if the predetermined variables made significant jumps at the breakpoints.

## Results

### Descriptive analysis

The reimbursement expenses of retired population were higher, and there was an obvious difference in expenses of inpatient. 84.5% retired population were aged 60 and above, and there was some early retirement phenomenon. Mean years of education was 7.9 (junior high school), and retired group’s mean value was slightly higher. 90% was married, and only a few was separated, divorced, widowed or never married. 52% chose to live with their children. Household assets of retired population was slightly higher than working group because of wealth accumulation. And the most were living in rural areas, but most retired population were in urban areas. Both the incidence and number of outpatient visits of the retired was slightly higher than the working. The number of chronic diseases, self-rated health status, the incidence of disability and BMI index were higher in the retired group, but the CES-D scores was higher in working group (Table 1).

**Table 1:** Descriptive analysis of variables

Variables	Full		Retirement		Working	
	N	Mean/%	N	Mean/%	N	Mean/%
reimbursement expenses (outpatient)	9925	0.366	1363	0.606	8384	0.305
reimbursement expenses (inpatient)	1080	7.414	237	8.593	750	6.9
reimbursement expenses (medicine)	25,987	0.302	3752	0.751	21,375	0.207
Age > 60	30,899	52.5	4265	84.5	25,044	45.8
Years of Education	30,881	7.865	4265	9.205	25,041	7.611
Marital status (1 = Married)	30,882	90.1	4265	91.8	25,042	90.1
Living with children (1 = with)	17,729	52.4	2854	44.7	13,956	54.1
household Assets Status(ln)	30,651	6.265	4251	7.233	24,997	6.059
Urban–rural (1 = urban)	23,910	24	3266	78.5	19,245	11.9
outpatient incidence (1 = receiving outpatient care)	30,576	16.7	4265	18.7	24,897	16.3
Number of outpatient visits	28,185	0.388	3609	0.499	23,184	0.371
Working hours	7882	51.365	468	47.695	7217	51.557
Number of chronic diseases	30,568	1.083	4237	1.5	24,873	1.013
Disability status (1 = disability)	17,931	9.7	2640	12.2	14,387	9
Self-rated health status	29,285	3.121	4081	3.134	23,881	3.117
CES-D Scores	65,458	8.112	7681	6.336	53,625	8.427
BMI	42,940	0.441	4808	0.528	36,337	0.427

### Basic regression results

**Fig. 1:**
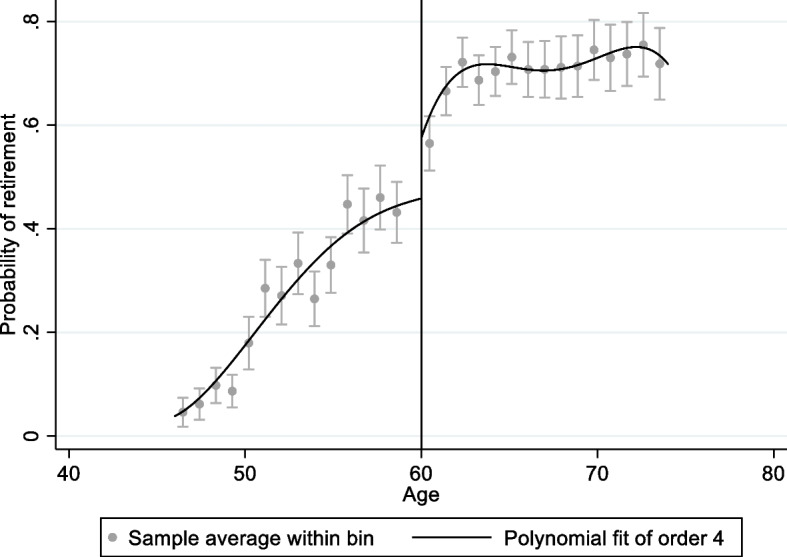
Trends of retirement rates

**Fig. 2:**
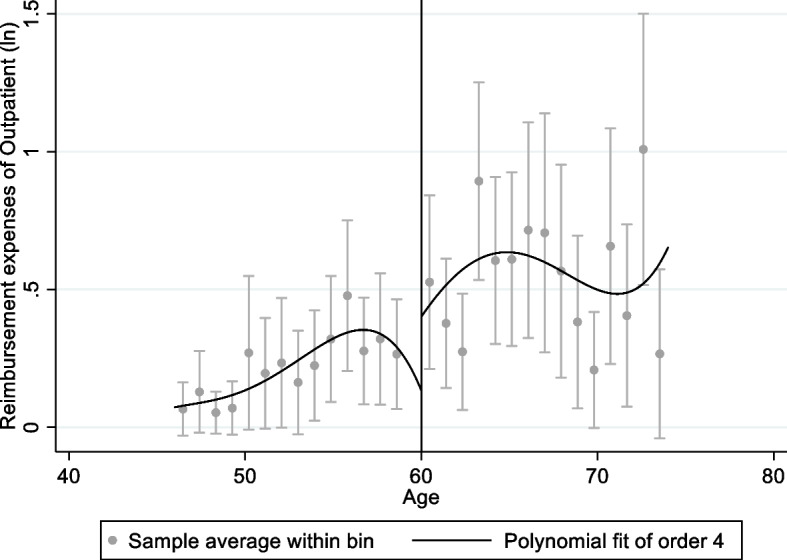
Medical reimbursement expenses of outpatient

**Fig. 3:**
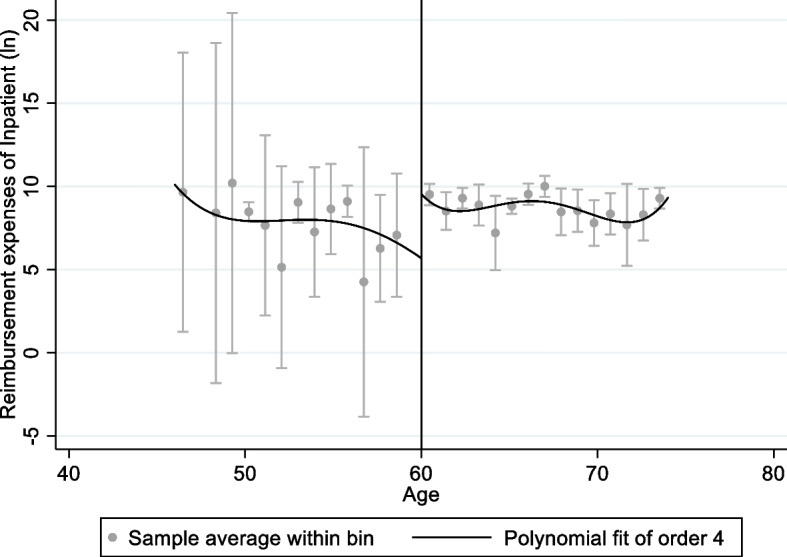
Medical reimbursement expenses of inpatient

**Fig. 4:**
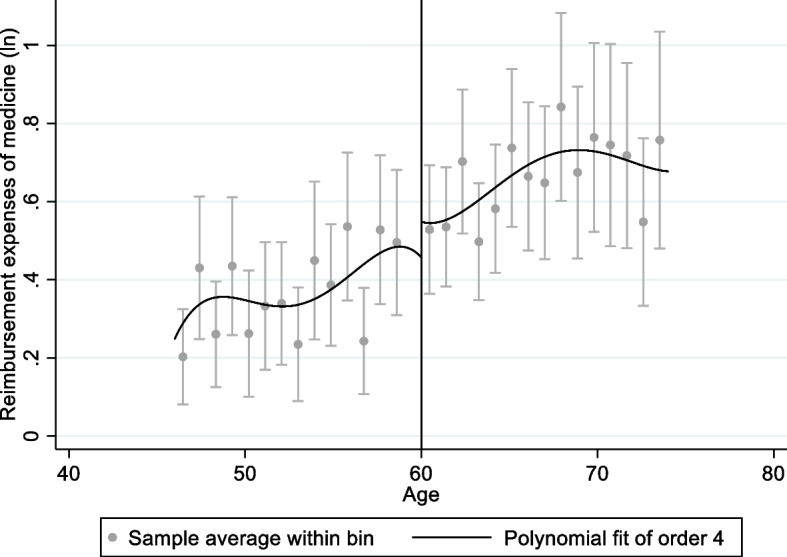
Medical reimbursement expenses of inpatient

Figures 1, 2, 3 and 4 shows graphical presentation of the dependent variables at the breakpoints. We found that there was an obvious difference at the breakpoint (age at 60) and the reimbursement expenses after retirement was higher than that before 60. Table 2 shows the effect of retirement on medical reimbursement expenses estimated by FRD, including reimbursement expenses of outpatient (model 1 ~ 2), reimbursement expenses of inpatient (model 3 ~ 4) and reimbursement expenses of medicine (model 5 ~ 6). It was found that retirement increased the reimbursement expenses of outpatient significantly and the local average treatment effect was 4.768 (*P* < 0.05). The estimation under different bandwidths was in Supplement.

**Table 2:** The effect of retirement on medical reimbursement expenses

Variable	Reimbursement expenses (outpatient)		Reimbursement expenses (inpatient)		Reimbursement expenses (medicine)	
	(1)	(2)	(3)	(4)	(5)	(6)
retirement	4.768** (2.239)	10.618 (10.515)	1.361 (7.008)	-42.660 (108.167)	0.081 (0.693)	-0.234 (1.554)
bandwidth	5	5	5	5	5	5
order	1	2	1	2	1	2
Control variables	YES	YES	YES	YES	YES	YES
*N*	4558	4558	639	639	14,228	14,228

①*p < 0.10, ** *p* < 0.05, *** *p* < 0.01

②Standard errors are in parentheses

③The results under different bandwidth were in supplement

④The triangular kernel function was used nonparametric estimation

⑤Age, square of age, years of education, marital status, living with children, and household assets were control variables

In addition, demographic characteristics and family-level factors were also associated with reimbursement expenses. There was a positive effect of years of education on reimbursement expenses of outpatient. It was possible that males with higher levels of education tended to have more health knowledge and self-regulatory behaviors, increasing medical services utilization [7]. And the expenses of outpatient among those living with children also increased significantly, and more household assets increased the reimbursement expenses of outpatient.

### Robustness test

#### Validity test

**Fig. 5:**
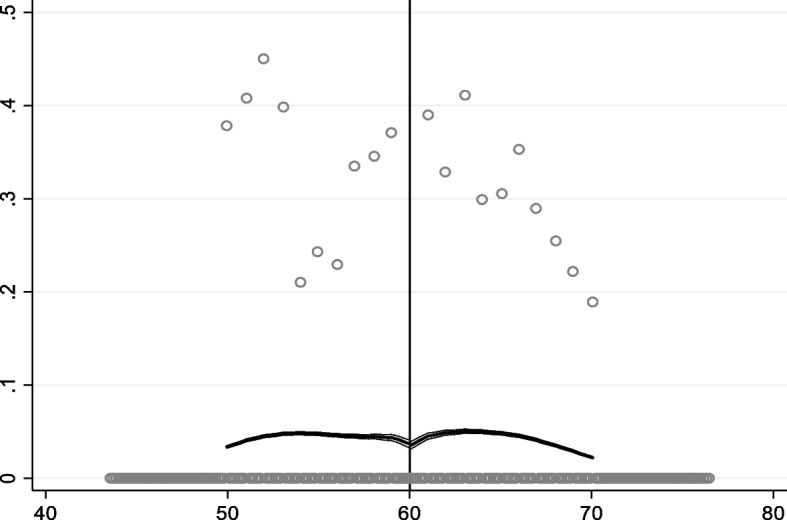
Density distribution of assignment variables around breakpoints

Fuzzy regression discontinuity needed to satisfy two requirements, (1) assignment variables could not be manipulated and, (2) the predetermined variable did not jump significantly at the breakpoint. On the one hand, according to McCrary’s [15], we examined assignment variable’ continuity at the breakpoint. Both the density function and the confidence interval almost overlapped at the breakpoint in Fig. 5. It shows that the difference before and after the breakpoint was -0.052 (SD = 0.084). Therefore, the assignment variable was continuously and smoothly distributed around the breakpoint, and there was no manipulation.

On the other one hand, the continuity of the predetermined variables was also tested. Table 3 shows that the significance level of each covariate could not reject the hypothesis of covariate continuity (*P* > 0.1). Each covariate did not significantly jump at the breakpoints and satisfied the continuity of the predetermined variables.

**Table 3:** Predetermined variable continuity test

Control variables	Coef	Std.Err	Z	P > Z	[95%Conf	Interval]
Years of education	0.368	0.368	1.00	0.318	-0.354	1.090
Marital Status	0.025	0.024	1.05	0.295	-0.022	0.073
Living with children	-0.022	0.062	-0.35	0.723	-0.143	0.099
Household Assets	0.143	0.288	0.50	0.619	-0.422	0.708
lwald	0.161	0.107	1.51	0.132	-0.048	0.371

#### Placebo test

The fictitious policy time was used to perform placebo test. Age at 55 and 65 were chosen as breakpoints to test whether retirement increased males’ medical reimbursement expenses under age at 55 and 65. Tables 4 shows that retirement did not increase medical imbursement expenses when 55 and 65 were set as retirement age. The placebo test supported the positive effect of retirement on reimbursement expense of outpatient.

**Table 4:** The placebo test

Variables	Breakpoint at 55			Breakpoint at 65		
	(7)	(8)	(9)	(10)	(11)	(12)
	Reimbursement expenses (outpatient)	reimbursement expenses (inpatient)	reimbursement expenses (medicine)	reimbursement expenses (outpatient)	Reimbursement expenses (inpatient)	Reimbursement expenses (medicine)
retirement	-0.072 (1.040)	2.558 (13.184)	-0.231 (0.508)	-16.851 (75.241)	-5.955 (10.181)	46.692 (1659.648)
Bandwidth	45	45	45	45	45	45
Order	1	1	1	1	1	1
Control variables	YES	YES	YES	YES	YES	YES
*N*	4568	640	14,380	4568	640	14,380

①*p < 0.10, ** *p* < 0.05, *** *p* < 0.01

②Standard errors are in parentheses

③The triangular kernel function was used nonparametric estimation

④Age, square of age, years of education, marital status, living with children, and household assets were control variables

#### Eliminate the bias of policy change

The expenses that were not reimbursed before can be reimbursed nowadays with the expansion of scope and proportion of reimbursement under China medical insurance reform, which biased the effect of retirement by leading to a sudden increase in the demand for medical services. To address this, the estimation was limited in period (before 2016) that medical reimbursement policy did not change. Table 5 shows that retirement increased the reimbursement expenses of outpatient significantly in 2011 ~ 2013 and 2011 ~ 2015 (*P* < 0.001), which was consistent with the results in Table 2.

**Table 5:** The effect of retirement on medical reimbursement expenses in different period

Variables	2011 ~ 2013			2011 ~ 2015		
	(13)	(14)	(15)	(16)	(17)	(18)
retirement	1.938*** (0.474)	2.096 (4.735)	0.928*** (0.353)	2.320*** (0.586)	6.667 (4.750)	0.386* (0.230)
Control variables	YES	YES	YES	YES	YES	YES
*N*	3868	221	6605	4282	439	12,055

①*p < 0.10, ** *p* < 0.05, *** *p* < 0.01

②Standard errors are in parentheses

③Model 13 and 16 were the estimation of reimbursement expenses of outpatient, model 14 and 17 were reimbursement expenses of inpatient and model 15 and 18 were reimbursement expenses of medicine

④The triangular kernel function was used nonparametric estimation

⑤Age, square of age, years of education, marital status, living with children, and household assets were control variables

#### Different age ranges

Different age ranges were associated with different optimal bandwidths when using FRD. The age ranged 45 ~ 75 years and 50 ~ 70 years were selected to increase the robustness. Table 6 shows the effect of retirement on medical reimbursement expenses under different age ranges. It was found that retirement increased reimbursement expenses of outpatient significantly among 45 to 75-year-old and 50 to 70-year-old group.

**Table 6:** The effect of retirement on medical reimbursement expenses in different group

Variables	45 ~ 75			50 ~ 70		
	(19)	(20)	(21)	(22)	(23)	(24)
Retirement	3.248*** (1.225)	5.799 (6.367)	0.334 (0.421)	3.248*** (1.225)	5.799 (6.367)	0.334 (0.421)
Control variables	YES	YES	YES	YES	YES	YES
*N*	4085	517	12,681	3221	387	9882

①*p < 0.10, ** *p* < 0.05, *** *p* < 0.01

②Standard errors are in parentheses

③Model 19 and 22 were the estimation of reimbursement expenses of outpatient, model 20 and 23 were reimbursement expenses of inpatient and model 21 and 24 were reimbursement expenses of medicine

④The triangular kernel function was used nonparametric estimation

⑤Age, square of age, years of education, marital status, living with children, and household assets were control variables

### Subgroup analysis

The heterogeneity of the relationship between retirement and reimbursement expenses was explored in this part under different education levels and marital status. Table 7 shows that retirement increased reimbursement expenses of outpatient and medicine significantly, and the increase of reimbursement expenses of outpatient was more significant in low-education group.

**Table 7:** The effect of retirement on medical reimbursement expenses by education level

Variables	High education			Low education		
	(25)	(26)	(27)	(28)	(29)	(30)
Retirement	0.909*** (0.322)	2.309 (1.498)	0.749*** (0.102)	1.575*** (0.295)	5.478 (4.567)	0.732*** (0.155)
Control variables	YES	YES	YES	YES	YES	YES
_cons	0.277 (0.291)	6.200*** (0.880)	0.146** (0.063)	0.151** (0.072)	5.013*** (0.821)	0.175*** (0.037)
*N*	894	223	5556	3679	427	8947

①*p < 0.10, ** *p* < 0.05, *** *p* < 0.01

②Standard errors are in parentheses

③Model 25 and 28 were the estimation of reimbursement expenses of outpatient, model 26 and 29 were reimbursement expenses of inpatient and model 27 and 30 were reimbursement expenses of medicine

④Age, square of age, years of education, marital status, living with children, and household assets were control variables

Table 8 shows that the effect of retirement on medical reimbursement expenses by marital status. It indicated that there was a significantly positive effect of retirement on reimbursement expenses of outpatient and medicine in those who were unmarried/divorced/widowed.

**Table 8:** The effect of retirement on medical reimbursement expenses by marital status

Variables	Married			Unmarried/divorced /widowed		
	(31)	(32)	(33)	(34)	(35)	(36)
Retirement	1.214*** (0.210)	4.455** (1.753)	0.727*** (0.083)	1.862** (0.931)	-4.677 (7.391)	1.316** (0.516)
Control variables	YES	YES	YES	YES	YES	YES
_cons	0.077 (0.075)	5.002*** (0.484)	0.104*** (0.030)	-0.059 (0.147)	6.387*** (1.158)	0.030 (0.066)
*N*	3918	538	12,601	655	112	1902

①*p < 0.10, ** *p* < 0.05, *** *p* < 0.01

②Standard errors are in parentheses

③Model 31 and 34 were the estimation of reimbursement expenses of outpatient, model 32 and 35 were reimbursement expenses of inpatient and model 33 and 36 were reimbursement expenses of medicine

④Age, square of age, years of education, marital status, living with children, and household assets were control variables

## Further analysis

### Time cost

Time cost was associated with the healthcare utilization, thus affecting the medical reimbursement expenses. In Table 9, the effect of retirement on outpatient incidence and outpatient times were explored first, and then the effect of retirement on medical reimbursement expenses were observed after controlling working hours in models after referring to Eibich [6]. Model 37 and 38 shows that retirement increased the outpatient incidence and outpatient times significantly, indicating that healthcare utilization increased after retirement. And when grouping working hours to do regression in model 39 and 40, it was found that retirement increased medical reimbursement expenses of outpatient significantly in those who worked more than 40 h before retirement. However, the effect was not found in less-than-40-h group.

**Table 9:** The effect of retirement on time cost

Variables	(37)	(38)	(39)	(40)
	Outpatient incidence	Outpatient times	reimbursement expenses (outpatient)(> = 40 h)	reimbursement expenses(outpatient) (< 40)
Retirement	0.175*** (0.026)	1.072*** (0.106)	2.700** (1.169)	13.600 (11.756)
Control variables	YES	YES	YES	YES
*N*	16,775	14,401	4413	148

①*p < 0.10, ** *p* < 0.05, *** *p* < 0.01

②Standard errors are in parentheses

③The triangular kernel function was used nonparametric estimation

④Age, square of age, years of education, marital status, living with children, and household assets were control variables

### Health status

The health status after retirement was explored in Table 10, including chronic diseases, activities of daily living (ADL), self-rated status, CES-D scores and BMI. Table 10 shows that retirement increased the number of chronic diseases, the risk of disability, CES-D scores and BMI index. And retirement decreased the self-rated health status significantly. Overall, retirement had a significant negative effect on males’ health status measured by subjective and objective health indicators.

**Table 10:** The effect of retirement on health status

Variables	(29)	(30)	(31)	(32)	(33)
	chronic diseases	ADL	Self-rated health status	CES-D	BMI
Retirement	1.673*** (0.094)	0.218*** (0.026)	-0.846*** (0.069)	1.192*** (0.395)	0.454* (0.253)
Control variables	YES	YES	YES	YES	YES
*N*	16,719	10,484	16,518	15,466	9826

①*p < 0.10, ** *p* < 0.05, *** *p* < 0.01

②Standard errors are in parentheses

③The triangular kernel function was used nonparametric estimation

④Age, square of age, years of education, marital status, living with children, and household assets were control variables

## Discussion

It was found that retirement increased reimbursement expenses of outpatient, and the result was robust through validity test, placebo test, eliminating the bias of policy change and estimation in different age ranges. Compared with previous studies focusing on healthcare utilization, the association between retirement and medical reimbursement expenses was investigated, because medical reimbursement in China was not for all diseases, and medical reimbursement expenses were not completely consistent with medical service demand. The association provided us another perspective to verify the growth of medical cost after retirement and enlightenment to optimize medical reimbursement policy.

The possible mechanism was low time cost and deteriorating health after retirement. On the one hand, more outpatient times and more reimbursement expenses of outpatient after retirement indicated that workers were not willing to sacrifice their working hours for healthcare utilization because of a high time cost, which was consistent with the findings of He et al. [9]. Low time cost after retirement motivated healthcare utilization, leading to an increase in medical reimbursement expenses of outpatient. On the other hand, retirement increased the number of chronic diseases, the risk of disability, CES-D scores and BMI index, and decreased the self-rated health status significantly. Retirement was seen as a stress or an important life event and implied a change of income and social status, resulting in some adverse health consequences [13, 20]. For example, individuals’ cognitive function decreased after retirement, particularly on males [12]. Also, retirement significantly increased depression index by 0.485% and significantly decreased self-rated health status by 6% [25]. There also was the same effect in physical health, as Behncke [1] found that retirement increased the risk of chronic diseases, and Godard [8] found that the incidence of obesity was significantly higher in males after retirement. Besides, Feng et al. [7] found that males’ weight and BMI index increased because of alcohol consumption and less physical activities.

There were group differences in the effect of retirement on medical reimbursement expenses of outpatient. Those with lower education had more reimbursement expenses of outpatient and medicine than those with higher education. It was possible that, first, males with low education might have less self-regulatory behaviors of health. For example, Feng et al. [7] found the males with low education had more drinking behaviors, resulting in poor health status after retirement Secondly, the low-education group was more likely to receive unreasonable medical services and healthcare utilization [26]. And those being divorced, widowed, or never-married had more reimbursement expenses of outpatient than being married. Possible explanations were that lacking spouses’ spiritual support and physical care reduced the health resources in older adults, inducing more medical demands.

## Conclusion

The effect of retirement on medical reimbursement expenses and its underlying mechanisms, and group heterogeneity were explored by using FRD based on the data from the China Health and Retirement Longitudinal Study (CHARLS) in 2011, 2013, 2015 and 2018. The findings were as follows,

First, retirement increased medical reimbursement expenses of outpatient. The result remained robust through validity test, placebo test, eliminate the bias of policy change and estimation in different age ranges.

Second, there were group differences in the effect of retirement on medical reimbursement by education level and marital status. Those with low education had more reimbursement expenses of outpatient than those with high education. Those being divorced, widowed, or never-married had more reimbursement expenses of outpatient than in married.

Finally, low time cost and deteriorating health status were the transmission mechanism in the effect of retirement on medical reimbursement expenses. It was found that working hours before retirement significantly decreased the outpatient incidence and outpatient times. Meanwhile, retirement had a negative effect on health status, including number of chronic diseases, activities of daily living, CES-D scores and BMI and self-rated health status.

The results of this study indicate that first, though delayed retirement policy reduces payment pressure by expanding contribution group and extending contribution years, but in the long run, the aging trend is irreversible, and the retired population will increase rapidly. It is difficult to achieve long-term effects by delaying the retirement age alone. Therefore, paying attention to health change before and after retirement, and taking measures to reduce health damage caused by retirement are advocated. Second, non-payment health insurance policy induces excessive utilization of medical services. It is important to make an adjustment to overcome unreasonable utilization. Finally, more healthcare utilization is not associated with actual health promotion. Health investment and healthy lifestyle are key factors in realizing old-age health.

## Supplementary Information

**Additional file 1: Supplement.** The effect of retirement on medical reimbursement expenses (under different bandwidth).

## Data Availability

The dataset analyzed was available from http://charls.pku.edu.cn/ after registration.

## Abbreviations

CHARLS: China health and retirement longitudinal study
RD: Regression discontinuity
FRD: Fuzzy regression discontinuity
LATE: Local average treatment effect

## Footnotes

1 Data Source: http://www.gov.cn/banshi/2005-08/04/content_20256.htm

2 Data Source: http://www.gov.cn/zhuanti/2015-06/13/content_2879014.htm

3 Data Source: http://www.gov.cn/zhengce/content/2016-01/12/content_10582.htm
