# Supplementary material for: Effect of retirement on medical reimbursement expenses—evidence from China
Source: Health Econ Rev. 2023 Apr 13;13:22. doi: 10.1186/s13561-023-00434-x (PMC10099839; doi:10.1186/s13561-023-00434-x)
Supplement: Supplementary file 1 — Additional file 1: Supplement. The effect of retirement on medical reimbursement expenses (under different bandwidth). [file 13561_2023_434_MOESM1_ESM.docx]

**Supplement**

The effect of retirement on medical reimbursement expenses (under different bandwidth)

|  | Reimbursement expenses (outpatient) | | Reimbursement expenses (inpatient) | | Reimbursement expenses (medicine) | |
| --- | --- | --- | --- | --- | --- | --- |
| variable |  |  |  |  |  |  |
| retirement | 3.2*** | 4.5** | 5.8 | 6.1 | 0.3 | 0.2 |
|  | (1.225) | (2.199) | (6.367) | (9.539) | (0.421) | (0.712) |
| bandwidth | 10 | 10 | 10 | 10 | 10 | 10 |
| order | 1 | 2 | 1 | 2 | 1 | 2 |
| Control variables | YES | YES | YES | YES | YES | YES |
| *N* | 4558 | 4558 | 639 | 639 | 14228 | 14228 |

Note：*p < 0.10, ** p < 0.05, *** p < 0.01；Standard errors are in parentheses. ③The triangular kernel function was used nonparametric estimation. ④Age, square of age, years of education, marital status, living with children, and household assets were control variables.
